# Supplementary figures and images for: Determinants of intention to improve oral hygiene behavior among students based on the theory of planned behavior: A structural equation modelling analysis
Source: PLoS One. 2021 Feb 25;16(2):e0247069. doi: 10.1371/journal.pone.0247069 (PMC7906382; doi:10.1371/journal.pone.0247069)

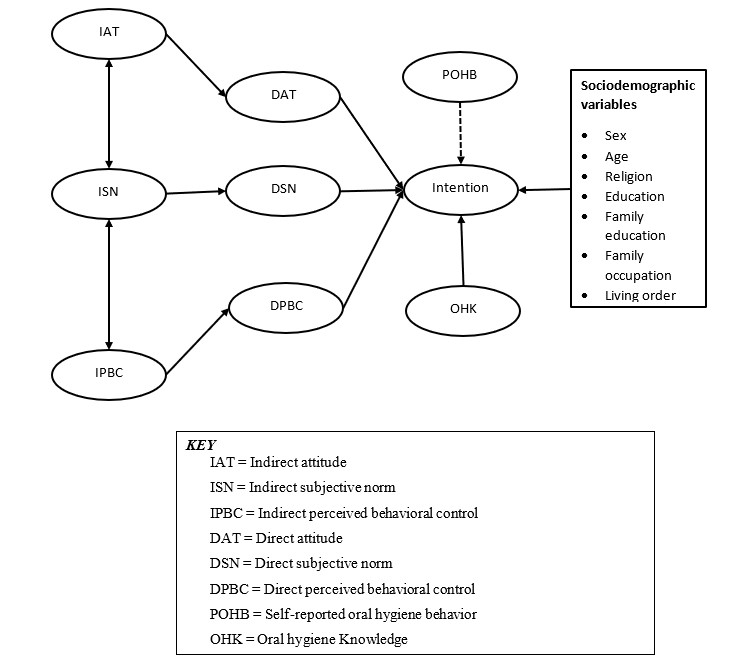

Supplement: S1 Fig — (TIF) [file pone.0247069.s001.tif]
