# Supplementary material for: Determinants of intention to improve oral hygiene behavior among students based on the theory of planned behavior: A structural equation modelling analysis
Source: PLoS One. 2021 Feb 25;16(2):e0247069. doi: 10.1371/journal.pone.0247069 (PMC7906382; doi:10.1371/journal.pone.0247069)
Supplement: Attachment — Submitted filename: PONE-D-20-32078.docx [file pone.0247069.s002.docx]

**Determinants of Intention to Improve Oral Hygiene Behavior Among Students Based on the Theory of Planned Behavior: A Structural Equation Modelling Analysis**

1- No extensive details are needed in study area (delete lines 98 to 101)
2- Check the academic year 2029/2020 in line 107
3- In exclusion criteria, why students transferred in the academic year not included in the study?
4- Did all students accept to participate in the present study??
5- How long did data collection take time? It should be mentioned in data collection section
6- Items of knowledge (OHK) should be described clearly in the data collection tool
7- Describe the eight items concerning OHB in the data collection tool
8- Line 179, 6 should be replaced by 7
9- Add table describing oral hygiene knowledge and self-reported oral hygiene behavior.
10- The discussion did not involve studies from developing countries
11- In the acknowledgement, you should acknowledge study participants (students).
