# Supplementary material for: Determinants of intention to improve oral hygiene behavior among students based on the theory of planned behavior: A structural equation modelling analysis
Source: PLoS One. 2021 Feb 25;16(2):e0247069. doi: 10.1371/journal.pone.0247069 (PMC7906382; doi:10.1371/journal.pone.0247069)
Supplement: Attachment — Submitted filename: Response to editor and reviewers OHBI PLOS ONE.docx [file pone.0247069.s003.docx]

Dear Mohammad Asghari Jafarabadi, editor and reviewers:

Hereby, we resubmit the enclosed –revised- manuscript ID PONE-D-20-32078 -[EMID:a6efc58a6a98f997], that is entitled “Determinants of Intention to Improve Oral Hygiene Behavior among Students Based on the Theory of Planned Behavior: A Structural Equation Modelling Analysis” to your journal*.*

We have read the comments carefully and we were able to implement all of them (see manuscript with track changes). While reading the manuscript critically, we spotted English grammar errors, which we and a language expert have corrected too. We hope that our revision will be felt as an improvement. We certainly feel this manuscript has improved thanks to the suggestions of the reviewers.

The datasets generated and/or analyzed in the current study are available at University of Gondar, College of Medicine and Health Science, Institute of Public Health in hard and soft copy repository [www.UoG.edu.et].

**Response to reviewer 1 comments and suggestions**

This manuscript is to determine/predict the behavior of students based on the theory of planned behavior (i.e., to predict an individual’s intention to engage in a behavior at a specific time and place). It also intends to use the structural equation modelling analysis to show the structural relationship by using a combination of factor analysis and multiple regressions, which basically should measure the relationship between the measured variables and the latent construct. Though, the topic seems an interesting one, the manuscript needs to be worked on by authors before it could be considered by this journal.

1. There is the need for the manuscript to be edited by a native speaker since there is a lot of grammatical issues and typographical errors, for example, see line 107 where you typed 2029/2020

***Response:*** Thanks for this comment*.* We gave this manuscript to an English language expert, Mr. Fikadie, for an English language edition**.** Then the entire manuscript was edited for grammatical and typographical errors based on his suggestion and commentaries. And of course, 2029/2020 must be 2019/2020. Thank you.

2. **Introduction**: please include literature on how the TPB has been used to determine/predict behaviors of people in other parts of the world before you limit it to Africa, and then Eastern Africa, and then to Ethiopia before you justify your topic. As it stands now, the latter part of the introduction is more about the explanation of the TPB which will only need about a line in the manuscript.

**Response:** It is clear that the theory of planned behavior has been applied to the prediction of the range of behaviors/intentions. However, our initial objective was to find evidence that demonstrates the usefulness of theory in predicting oral hygiene behavior. In this regard, we did not find any published papers involving the application of TPB in the prediction of oral health behavior of students in Ethiopia, not even in Africa. For this reason, we did not describe the usefulness of TPB in Ethiopia or Africa. However, we have now added some evidence to demonstrate how useful TPB is in predicting different health behaviors other than oral hygiene behaviors in Ethiopia. We also tried to mention the usefulness of TPB in predicting various health behaviors worldwide. (**See manuscript, line 67 to 79**)

3. **Methods**: This is where i find most statistical flaws. Please explain further and clearly on how the sample size was arrived rather than the vague equations. Was it calculated on assumption (because of the 50%), if 'yes' that will mean there have been no study conducted so far on this area as far as Ethiopia is concerned. If 'No' then please cite the paper upon which you are basing your calculations with the sample that those authors used. Please show your exposure and dependent variables and how they will be measured on the methodology section.

**Response:** Unlike the sample sizes for means and proportions, which are obtained with a certain level of confidence from mathematical calculations, the sample sizes for the estimation of SEM are derived in another way. There is no single all fitted sample size calculation for SEM-based studies. As a result, there are various recommendations. However, it is taught that the required sample size for SEM depends on the complexity of the model, the relationship between the observed and latent variables, and the type I and II error. Some scholars use the rule of thumb and others use a kind of hypothesis to compute a sample for the SEM-based study. For our study, the sample size was calculated using a statistical calculator developed by Professor Daniel. This calculator calculates the required sample size as per the following assumptions.

	The number of observed and latent variables in the model,

	The anticipated effect size and

	The desired probability, and statistical power levels.

**Reference**: Soper, D.S. (2020). A-priori Sample Size Calculator for Structural Equation Models [Software]. Available from <https://www.danielsoper.com/statcalc>

We also mentioned the study sample size and number of variables used in the present study **(see in the manuscript, line 118-142).** Furthermore, how we measured each variable is already described in the “Data Collection and Measurement” sub-heading of the Methodology section. **(See in the manuscript in line 143-234)**

**4. Results:** Again, I would recommend that you do not categorize your variables so that you can explore your data. And this is where you would have to explore more, for example on socio-demographics, please show how many were 15 years and so on. Then report on Means, median, Standard deviations, and so on (please, read a similar manuscript like 'Åstrøm AN, Lie SA, Gülcan F. Applying the theory of planned behavior to self-report dental attendance in Norwegian adults through a structural equation modeling approach. BMC Oral Health. 2018') and report appropriately if you want to stick to the TPB.
Again on your results, I do not see how those variables you measured in Table 1 determines/predict the student's behavior as per the theory, for example how does 'Mother's educational status determine/predicts the oral hygiene of the student'. I also do not see any figure that is displaying the relationship between the regression and the variables. This is why you will need to redo the analysis so that you can well display the relationships between the Means Medians, SD, and others with your multiple regression. That will bring out the beauty and understanding of what you want people to know.

**Response:** The age range of the students is very narrow (16-24 years). Therefore, we did not rank the variable in more categories than we did. We have already mentioned the mean and standard deviation of the age of participants (**see line 268 of the manuscript**). Furthermore, the primary objective of this study was not to predict students' oral hygiene behavior, but their intention to engage in OHB. According to the TPB, the behavior should be measured after one week, one month, or three months, and so on depending on the nature of the behavior. However, as it is mentioned in the limitation section of our study, behavior and other TPB variables were measured at the same time. As a result, we did not consider past OHB as an outcome variable. Nevertheless, we used this variable as a predictor of the intention to engage in OHB in the future.

Moreover, we did an SEM analysis to confirm the hypothesized relationships supposed by the TPB. The extent to which one variable has an impact over the other variable is measured by the path coefficient that links those variables together. This estimate is analogous to a regression coefficient in the ordinary regression analysis. Moreover, the significance of the association is determined by the critical ratio and p-value of the estimate. All these things might be presented in diagrams or tables. In the previous manuscript, we presented the SEM result in diagrammatic form. In the revised manuscript, we have added a table containing the direct and indirect path coefficient with a 95% confidence interval to make it easier to understand. (**See the manuscript in table 6, line 355**)

**5.Discussion**: When you are done with the above, that would affect your discussions, and I would like to see how your work differs from others rather than just conforming to what is already done. Please discuss your own work for example on how ' mother's education determines the behavior of a child, the reasons behind that and many more. I strongly believe that when this is redone, you would have a lot to discuss looking at your variables, and your paper would stand out among the lots.

**Response:** We have made many changes to the discussion section of this manuscript. In particular, we discussed variables that were not significant predictors of OHBI in our study, but in earlier studies (**see** **line 398-413 of the manuscript**). We have tried to compare and contrast our results to what has already been done and provide possible explanations for conformities as well as differences. The current study is also the first to assess oral hygiene behavioral intention and its determinants among students in our country, where oral health practices are very poor in this population group. In addition to this, the study identified the most important psychosocial determinant of intention to perform OHB which is of paramount importance for behavioral change intervention aimed at improving the OHB of students. In the end, improve their oral and general health.

**Response to reviewer 2 comments and suggestions**

1- No extensive details are needed in the study area (delete lines 98 to 101)

**Response:** Thank you, we have deleted these sentences (**see in manuscript with track changes in lines 115-118**)

2- Check the academic year 2029/2020 in line 107

**Response:** Sorry for this mistake, we have changed this in 2019/2020 (**See in the manuscript in line 107**)

3- In exclusion criteria, why students transferred in the academic year not included in the study?

**Response:** We excluded students who were transferred within the school year at the time the data was collected. We have done this due to the behavior/intention towards oral hygiene varies according to residence and culture in our facility. For example, oral hygiene behavior is extremely low in rural residents compared with the urban population. Furthermore, according to Planned Behavior Theory (BPT), the intention of the student is determined by the student's attitude, subjective standard, and perceived behavioral control of oral hygiene behavior. In this respect, students from different societies may have different attitudes, subjective norms, perceived control, and intentions which may affect the associations between the TPB variable. Moreover, these students are not well adapted to the social system i.e. they may act as what they behaved in their origin or may overact because of over-expectation of living in cities, both affects our outcome of interest. From this point of view, it is difficult to draw any conclusion from data containing information from students who are not representative of the city's students. Thus, we excluded those students from the study to avoid under /overestimation of the study’s result.

4- Did all students accept to participate in the present study??

**Response:** No. As we have mentioned in the result section of the abstract and main text, 393 (97.5%) of the students participated from the estimated sample size i.e. 403. (**See the manuscript in line 34 and 267**)

5- How long did data collection take time? It should be mentioned in the data collection section

**Response:** Alright, we have mentioned the data collection period **in line 144 in the manuscript** under the data collection section.

6- Items of knowledge (OHK) should be described clearly in the data collection tool

**Response:** All items used to measure oral hygiene knowledge are depicted in the result section (**see Table 2 in line 280-281 in the manuscript).** Thus, we didn’t list those items in the data collection tool just not to make things redundant.

7- Describe the eight items concerning OHB in the data collection tool

**Response:** All items concerning OHB are depicted in the result section (see in table 3 **in line 282-283 in the manuscript).** Thus, we didn’t list those items in the data collection tool just not to make things redundant.

8- Line 179, 6 should be replaced by 7

**Response:** It was a typological error we made and correction was made accordingly **(see in line 216 in the revised manuscript with track changes)**

9- Add table describing oral hygiene knowledge and self-reported oral hygiene behavior.

**Response:** A table describing oral hygiene knowledge and self-reported oral hygiene behavior was added in the result section (**see the revised manuscript with track change in line 310 (table 2) and line 312 (table 3)** **respectively)**.

10- The discussion did not involve studies from developing countries

**Response:** Yeah, you are right. That was also our concern. Unfortunately, to the best of our knowledge, there are no published studies concerning the determinants of intention to oral hygiene behavior in those countries.

11- In the acknowledgment, you should acknowledge study participants (students).

**Response:** Thank you. Correction was made upon your comment (**See revised manuscript with track changes in line** **511 to 513).**

**Response to Reviewer 3 comments and Suggestions**

A well written manuscript with intelligent inferences drawn from the statistical analysis. The methodology is sound and the research area is of importance in its relevant field. Behavioural interventions have been increasingly recognized as effective preventive strategies in different domains of health. There were few grammatical mistakes detected in various components and a professional proof reading of manuscript is advised.

**Response:** Thank you. The manuscript reviewed for language by an English expert and revisions were made accordingly.
